# Risk of TB disease in individuals with cancer

**DOI:** 10.5588/ijtldopen.24.0440

**Published:** 2025-01-01

**Authors:** T. Diefenbach-Elstob, S. Tabrizi, P. Rivest, A. Benedetti, L. Azoulay, K. Schwartzman, C. Greenaway

**Affiliations:** ^1^ Centre for Clinical Epidemiology, Lady Davis Institute, Jewish General Hospital, Montreal, QC, Canada;; ^2^ Department of Medicine, McGill University, Montreal, QC, Canada;; ^3^ Département de médicine sociale et préventive, École de santé publique de l'Université de Montréal, Montréal, QC, Canada;; ^4^ Direction régionale de santé publique, Centre intégré universitaire de santé et de services sociaux du Centre-Sud-de-l'Île-de-Montréal, Montréal, QC, Canada;; ^5^ Department of Epidemiology, Biostatistics & Occupational Health, McGill University, Montreal, QC, Canada;; ^6^ Respiratory Division, Department of Medicine, McGill University, Montreal, QC, Canada;; ^7^ McGill International TB Centre, Montreal, QC, Canada;; ^8^ Montreal Chest Institute, Montreal, Canada;; ^9^ Research Institute of the McGill University Health Centre, Montreal, QC, Canada;; ^10^ Division of Infectious Diseases, SMBD-Jewish General Hospital, Montreal, QC, Canada.

**Keywords:** reactivation, malignancy, screening

## Abstract

**BACKGROUND:**

Cancer increases the risk of developing TB disease; however, there are limited data on the magnitude of risk by cancer type and timing after diagnosis of cancer in low TB incidence settings.

**METHODS:**

We conducted a nested case-control study of persons in Quebec between 1993 and 2017, including people with TB disease and matched controls. Conditional logistic regression was used to estimate adjusted odds ratios (aORs) of developing TB among people with cancer overall, by sub-type, and by time from cancer to TB diagnosis.

**RESULTS:**

There were 4,283 people with TB disease and 268,420 matched controls. The median age for people with TB disease and controls was respectively 46 years (IQR 30–67) and 36 years (24–47). Prior exposure to cancer was associated with TB disease (aOR 6.3, 95% CI 5.3–7.6). The risk of TB diagnosis was highest within 3 months of cancer diagnosis (aOR 26.6, 95% CI 19.6–36.2), with 60% of diagnoses of TB disease occurring within 6 months of cancer diagnosis.

**CONCLUSION:**

Risk of TB varies over time and by cancer type. Screening and treatment should be considered for potentially preventable TB (diagnosed more than 6 months post-cancer), particularly in those with respiratory, haematologic, and head and neck cancers.

## RÉSUMÉ

**CONTEXTE:**

Le cancer augmente le risque de développer une TB ; cependant, il existe peu de données sur l'ampleur du risque par type de cancer et le moment après le diagnostic de cancer dans les contextes à faible incidence de la TB.

**MÉTHODES:**

Nous avons mené une étude cas-témoins emboîtée auprès de personnes au Québec, Canada, entre 1993 et 2017, y compris des personnes atteintes de TB et des témoins appariés. La régression logistique conditionnelle a été utilisée pour estimer les rapports de cotes ajustés (aOR) de développer une TB chez les personnes atteintes de cancer dans l'ensemble, par sous-type et par temps écoulé entre le cancer et le diagnostic de la TB.

**RÉSULTATS:**

Il y avait 4 283 personnes atteintes de tuberculose et 268 420 témoins appariés. L'âge médian des personnes atteintes de TB et des témoins était respectivement de 46 ans (IQR 30–67) et de 36 ans (24–47). Une exposition antérieure au cancer était associée à la tuberculose (aOR 6,3 ; IC à 95% 5,3–7,6). Le risque de diagnostic de TB était le plus élevé dans les 3 mois suivant le diagnostic de cancer (aOR 26,6 ; IC à 95% 19,6–36,2), 60% des diagnostics de TB survenant dans les 6 mois suivant le diagnostic de cancer.

**CONCLUSION:**

Le risque de TB varie dans le temps et selon le type de cancer. Le dépistage et le traitement de la TB potentiellement évitable (diagnostiquée plus de 6 mois après le cancer), en particulier chez les personnes atteintes de cancers respiratoires, hématologiques et de la tête et du cou, doivent être envisagés.

Cancer is an important risk factor in the development of TB disease. Most studies describing the risk of developing TB disease after cancer have been conducted in intermediate and high TB incidence settings.^1–8^ Studies in low TB incidence settings have found a wide range of TB risks for specific cancer types, and there are limited data on the timing of TB after each cancer type.^9–11^ In a recent systematic review and meta-analysis, the risk of development of TB disease was 26, 16 and 9-fold higher in patients with hematologic, head, neck, and lung cancers in the United States, but the timing of TB after cancer could not be determined.^9^

A retrospective cohort study among foreign-born persons in British Columbia, Canada, described an increased risk of TB within 5 years post-cancer diagnosis, with the hazard of developing TB highest for lung, sarcoma, haematological and gastrointestinal cancers.^10^ A Danish cohort study found the highest risk of developing TB occurred in the first year post-cancer diagnosis, but cancer-type-specific risks were not reported.^6^

The Canadian Tuberculosis Standards recognise cancer as an important risk factor for the development of TB disease,^12^ but there are limited data about which cancers pose the highest risk and the time at greatest risk. In this study, we describe cancer-specific risk and timing of TB disease within 2 years of cancer diagnosis among people living in a low TB incidence setting in Quebec, Canada.

## METHODS

### Study design and linked administrative cohort

This was a nested case-control study of persons with and without TB registered for health care in Quebec during 1993–2017. All diagnoses of TB disease in Quebec between 1 January 1993 and 31 December 2017, identified in the reportable disease database, were linked to provincial administrative datasets. In Quebec, nominal reporting of TB disease is mandatory for treating clinicians and laboratories making microbiologic diagnoses.

Databases were linked deterministically (1987–2018) through a unique healthcare number to the provincial health insurance registry (*Régie de l'assurance maladie du Québec-Fichier d'inscription des personnes assurées*, RAMQ-FIPA), physician billings, pharmaceutical services, hospitalisations, and the Quebec Cancer Registry (RQC). Deaths were ascertained through linkage with the death registry (1990–2015) and RAMQ-FIPA (2016–2018). The landed immigration database was linked deterministically (1980–2018) with a visa number to RAMQ-FIPA. Before 2011, cancer cases in RQC were identified from hospital admissions, day surgery and the Quebec death registry. Since 2011, cancer diagnoses from pathology reports have been added, capturing diagnoses from outpatient settings.

### TB cases and controls

Based on the database linkages, potential cases and controls were stratified by immigrant status (immigrant versus non-immigrant). Cases included people with TB disease identified in the reportable disease database, diagnosed during 1993–2017, and registered in the provincial health insurance registry with at least 1 year of health coverage before TB diagnosis. TB cases identified as foreign-born in the reportable diseases data that were not linked to the landed immigration database were excluded. Controls registered in the health insurance database on the date of the TB diagnosis of the case were selected and matched based on the same immigration status and health data available for the same lookback period as the case. We matched 80 immigrant controls per immigrant case and 50 non-immigrant controls per non-immigrant case. Data were extracted for up to 2 years before and after the TB diagnosis date for cases and up to two years before the index date for matched controls.

### Cancer and covariate definitions

Demographic variables were extracted from the health insurance registry. Age was calculated based on the date of TB diagnosis, and the same calendar date was used for each control. Medical comorbidities (HIV, diabetes, chronic dialysis, and transplant) were identified from health administrative data during the lookback period using algorithms based on standard definitions, published studies, and expert advice (Supplementary Data 1).^13–21^

Quebec’s cancer registry classifies malignant cancers using the International Classification of Diseases for Oncology, 3^rd^ Edition (ICD-O-3) codes. Cancers were identified by the biological site of the primary malignancy and divided into solid and hematologic categories and sub-categories based on standard definitions, as well as published reports (Supplementary Data 2).^22,23^ Non-melanoma skin cancers were excluded as they were not reported to the Quebec cancer registry in 2011.

Co-prevalent TB and cancer diagnoses were defined as TB diagnoses occurring within three months before or after cancer diagnosis. Potentially preventable TB (TB which may be prevented through latent TB screening and treatment at the time of cancer diagnosis) was defined as TB diagnosed more than 6 months following cancer diagnosis.

### Statistical analysis

Data for people with TB disease and controls were pooled for analysis. Demographic and clinical characteristics were tabulated, and the number and proportion of individuals with a cancer diagnosis were determined overall and by type. The timing of TB disease in relation to cancer was plotted for all individuals diagnosed with both TB and cancer. Conditional logistic regression was used to estimate odds ratios (ORs) for TB disease among people with a diagnosis of any cancer (vs no cancer) by cancer type (solid or haematologic vs no cancer, and solid or haematologic subtype vs no cancer), and by time from cancer to TB diagnosis (vs no cancer). ORs were adjusted for age, sex, and medical comorbidities. Due to differences in the age structure of people with TB disease and controls, sensitivity analyses included models with additional adjustments for age (Supplementary Data 4 and 5). Analyses were undertaken in SAS v9.4 (SAS Institute Inc, Cary, NC, USA).

### Ethics

Study approval was granted by the *Commission d’accès du Québec*, Montreal, QC, Canada, and the research ethics committee of the Integrated Health and Social Services University Network for West-Central Montreal, QC, Canada.

## RESULTS

The study included 272,703 people, comprising 4,283 (1.6%) with TB and 268,420 matched controls. The median lookback was 2 years. People with TB disease had a higher median age (46 vs 36 years) and proportion of males (56.1% vs 50.1%) than controls (Table 1). Immigrant cases and controls were younger than non-immigrant cases and controls (median age: 38 and 36 vs 57 and 37 years). Compared with controls, a higher proportion of people with TB disease had medical comorbidities, including HIV, diabetes and end-stage kidney disease on chronic dialysis (Table 1).

**Table 1.** Demographic and clinical characteristics of people with TB disease and controls.

		Total *n* (%)	TB disease *n* (%)	Controls *n* (%)
Total		272,703 (100.0)	4,283 (1.6)	268,420 (98.4)
Sex	Female	135,954 (49.9)	1,879 (43.9)	134,075 (49.9)
	Male	136,749 (50.1)	2,404 (56.1)	134,345 (50.1)
Age, years, median [IQR]		36 [24–47]	46 [30–67]	36 [24–47]
Age group, years*	0–14	30,236 (11.1)	261 (6.1)	29,975 (11.2)
	15–24	38,688 (14.2)	468 (10.9)	38,220 (14.2)
	25–34	55,637 (20.4)	638 (14.9)	54,999 (20.5)
	35–44	65,643 (24.1)	691 (16.1)	64,952 (24.2)
	45–54	41,789 (15.3)	505 (11.8)	41,284 (15.4)
	55–64	22,814 (8.4)	498 (11.6)	22,316 (8.3)
	65–74	11,946 (4.4)	541 (12.6)	11,405 (4.2)
	≥75	5,950 (2.2)	681 (15.9)	5,269 (2.0)
Index period	1993–1999	98,301 (36.0)	1,581 (36.9)	96,720 (36.0)
	2000–2009	95,637 (35.1)	1,497 (35.0)	94,140 (35.1)
	2010–2017	78,765 (28.9)	1,205 (28.1)	77,560 (28.9)
TB disease site	Pulmonary	—	2,867 (66.9)	—
	Extrapulmonary	—	1,198 (28.0)	—
	Concurrent	—	213 (5.0)	—
	Missing/Unknown	—	5 (0.1)	—
HIV^†^	No	272,218 (99.8)	4,109 (95.9)	268,109 (99.9)
	Yes	485 (0.2)	174 (4.1)	311 (0.1)
Diabetes^†^	No	264,882 (97.1)	3,831 (89.4)	261,051 (97.3)
	Yes	7,821 (2.9)	452 (10.6)	7,369 (2.7)
Chronic dialysis^†^	No	272,602 (100)	4,252 (99.3)	268,350 (100)
	Yes	101 (0.0)	31 (0.7)	70 (0.0)
Transplant^†^	No	272,657 (100)	4,273 (99.8)	268,384 (100)
	Yes	46 (0.0)	10 (0.2)	36 (0.0)
Cancer	No	271,589 (99.6)	4,091 (95.5)	267,498 (99.7)
	Yes	1,114 (0.4)	192 (4.5)	922 (0.3)
Time from cancer to TB diagnosis^‡^	Cancer not identified	271,589 (99.6)	4,091 (95.5)	267,498 (99.7)
	Up to 3 months	216 (0.1)	102 (2.4)	114 (0.0)
	4–6 months	141 (0.1)	14 (0.3)	127 (0.1)
	7–12 months	265 (0.1)	34 (0.8)	231 (0.1)
	13–18 months	255 (0.1)	19 (0.4)	236 (0.1)
	19–24 months	237 (0.1)	23 (0.5)	214 (0.1)

* Age at the date of the TB diagnosis for the person with TB disease, and on the same date for each matched control;

† Medical comorbidities identified during the lookback period for cases and controls;

‡ Time from cancer to the date of diagnosis of TB for the person with TB disease, and time from cancer to the TB diagnosis date of the matched case for each control.

IQR = interquartile range.

There were 1,114 (0.4%) individuals diagnosed with cancer during the lookback period, including 192/4,283 (4.5%) among people with TB disease and 922/268,420 (0.3%) among controls. Most cancer sub-types were solid cancers (1,005/1,114; 90%), and 109 (10%) were haematologic malignancies (Table 2). The most common cancers among people with TB disease were respiratory (*n* = 59, 1.4% vs *n* = 33, 0.01% among controls) and haematologic malignancies (*n* = 37, 0.9% vs *n* = 72, 0.03%) (Supplementary Data 3). Among 192 people with TB disease and cancer during the lookback period, the median time from cancer to TB diagnosis was 2.6 months (IQR 1.2–10.4) (Table 2). The median time to TB diagnosis varied and was shortest for leukaemia (1.1 months, IQR 0.8–4.8), respiratory (1.6 months, IQR 1.0–7.1), lymphoma (2.2 months, IQR 1.3–12.2), and male genital system (2.6 months, IQR 1.1–9.8) cancers. Most people were diagnosed with TB (*n* = 116, 60.4%) within 6 months of cancer diagnosis. There were many co-prevalent TB diagnoses among people with respiratory cancer, with 66 TB diagnoses within 3 months before or after cancer diagnosis. Figure 1 shows the timing of TB diagnosis relative to cancer diagnosis for different cancer types.

**Table 2.** Characteristics of cancer among all people with TB disease and controls.

	Controls (*n* = 268,420) *n* (%)	People with TB disease (*n* = 4,283) *n* (%)	Time from cancer to TB diagnosis, months Median [IQR]	TB diagnoses occurring 0–3 months before a cancer diagnosis *n*	TB diagnoses occurring after a cancer diagnosis			
					0–3 months *n* (%)	4–6 months *n* (%)	7–12 months *n* (%)	13–24 months *n* (%)
Any cancer	922 (0.34)	192 (4.5)	2.6 [1.2–10.4]	63	102 (53.1)	14 (7.3)	34 (17.7)	42 (21.9)
Solid cancer types	850 (0.32)	155 (3.6)	2.8 [1.2–10.2]	53	81 (52.3)	10 (6.5)	31 (20.0)	33 (21.3)
Breast	210 (0.08)	9 (0.21)	8.7 [4.7–9.5]	2	2 (22.2)	2 (22.2)	4 (44.4)	1 (11.1)
Gastrointestinal	115 (0.04)	28 (0.65)	3.9 [1.0–15.5]	10	12 (42.9)	3 (10.7)	2 (7.1)	11 (39.3)
Gynaecological	153 (0.06)	6 (0.14)	8.5 [2.2–12.2]	5	2 (33.3)	0 (0.0)	2 (33.3)	2 (33.3)
Head and neck	25 (0.01)	9 (0.21)	6.4 [1.2–11.7]	4	3 (33.3)	1 (11.1)	3 (33.3)	2 (22.2)
Male genital system	116 (0.04)	14 (0.33)	2.6 [1.1–9.8]	3	9 (64.3)	0 (0.0)	3 (21.4)	2 (14.3)
Other solid	198 (0.07)	30 (0.70)	6.8 [1.9–12.3]	5	11 (36.7)	3 (10.0)	8 (26.7)	8 (26.7)
Respiratory*	33 (0.01)	59 (1.4)	1.6 [1.0–7.1]	24	42 (71.2)	1 (1.7)	9 (15.3)	7 (11.9)
Haematologic cancer types	72 (0.03)	37 (0.86)	2.0 [1.1–11.0]	10	21 (56.8)	4 (10.8)	3 (8.1)	9 (24.3)
Leukaemia	13 (0.00)	8 (0.19)	1.1 [0.8–4.8]	5	6 (75.0)	0 (0.0)	1 (12.5)	1 (12.5)
Lymphoma	45 (0.02)	16 (0.37)	2.2 [1.3–12.2]	2	9 (56.3)	2 (12.5)	1 (6.3)	4 (25.0)
Other haematologic*	14 (0.01)	13 (0.30)	3.8 [1.2–13.7]	3	6 (46.2)	2 (15.4)	1 (7.7)	4 (30.8)

* Respiratory includes cancers coded as bronchus and lung, trachea, mediastinum and pleura, other and ill-defined sites within respiratory system and intrathoracic organs; other haematologic includes multiple myeloma, mast cell tumours, excluding mast cell leukaemia, neoplasms of histiocytes and accessory lymphoid cells, malignant immunoproliferative diseases, chronic myeloproliferative disorders (except chronic neutrophilic leukaemia and chronic eosinophilic leukaemia), other haematologic disorders, myelodysplastic syndromes.

IQR = interquartile range.

**Figure.**
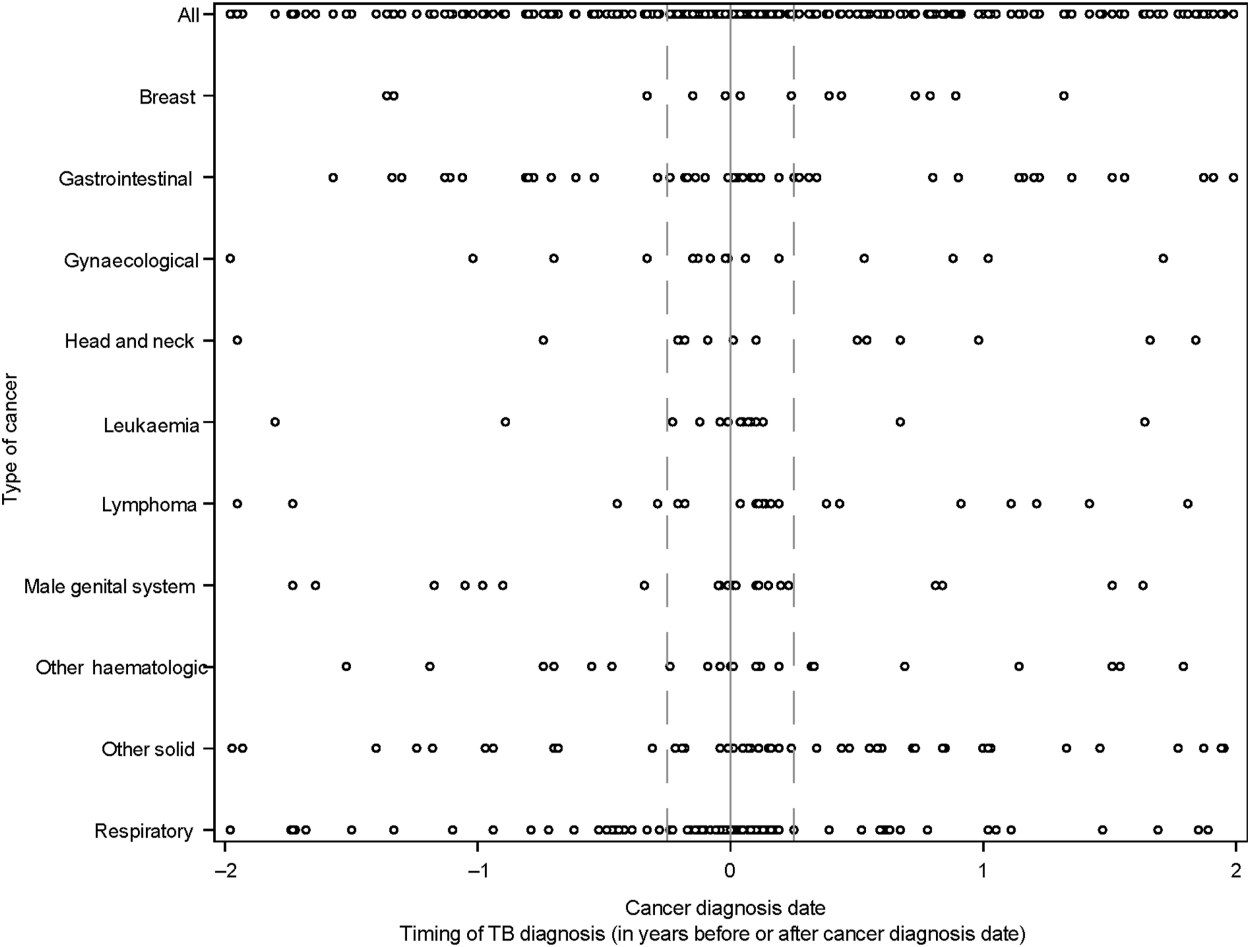
Timing of diagnoses of TB disease (number of years before or after cancer diagnosis) for individuals with both a TB and a cancer diagnosis. The hatched line indicates the point 3 months before and after the cancer diagnosis date (note: for each person, the look-forward duration used was the same as their lookback duration, i.e. 1–2 years).

Prior exposure to any cancer was associated with a higher risk of developing TB disease, with an aOR of 6.3 (95% CI 5.3–7.6). After stratification by time from cancer diagnosis, the greatest risk of TB was in the first 3 months following cancer diagnosis (aOR 26.6, 95% CI 19.6–36.2). The risk remained elevated for the entire follow-up period (Table 3). Development of TB disease associated with prior exposure to specific cancer sub-types was greatest for respiratory cancers (aOR 47.7, 95% CI 29.0–78.4), followed by haematological (aOR range 11.5–20.8), head and neck (aOR 11.0, 95% CI 4.7–25.7), gastrointestinal (aOR 5.9, 95% CI 3.7–9.2), other solid (aOR 4.9, 95% CI 3.2–7.5), and male genital system (aOR 2.2, 95% CI 1.2–4.0) cancers (Table 4).

**Table 3.** Conditional logistic regression for odds of diagnosis of TB associated with prior exposure to cancer, adjusted for demographic characteristics and medical comorbidities.

Variable		cOR (95% CI)	Adjusted (for cancer) aOR (95% CI)	Adjusted (for time from cancer to TB) aOR (95% CI)
Cancer	No	Reference	Reference	—
	Yes	12.8 (10.9–15.0)	6.3 (5.3–7.6)	—
Time from cancer to TB diagnosis*	No cancer	Reference	—	Reference
	Up to 3 months	53.1 (40.6–69.5)	—	26.6 (19.6–36.2)
	4–6 months	6.8 (3.9–11.9)	—	3.9 (2.1–7.0)
	7–12 months	9.3 (6.4–13.3)	—	4.6 (3.1–6.9)
	13–18 months	4.9 (3.1–7.9)	—	2.1 (1.3–3.6)
	19–24 months	6.4 (4.2–9.9)	—	3.1 (2.0–5.0)
Sex	Female	Reference	Reference	Reference
	Male	1.3 (1.2–1.4)	1.3 (1.3–1.4)	1.3 (1.3–1.4)
Age group, years^†^	0–14	0.63 (0.54–0.74)	0.62 (0.54–0.73)	0.63 (0.54–0.73)
	15–24	Reference	Reference	Reference
	25–34	0.99 (0.88–1.1)	0.94 (0.83–1.1)	0.94 (0.83–1.1)
	35–44	0.92 (0.82–1.0)	0.83 (0.74–0.94)	0.83 (0.74–0.94)
	45–54	1.0 (0.90–1.2)	0.89 (0.78–1.0)	0.88 (0.78–1.0)
	55–64	1.9 (1.7–2.1)	1.6 (1.4–1.9)	1.6 (1.4–1.9)
	65–74	4.2 (3.7–4.7)	3.5 (3.0–3.9)	3.4 (3.0–3.9)
	≥75	12.3 (10.9–14.0)	10.5 (9.2–11.9)	10.5 (9.2–11.9)
HIV^‡^	No	Reference	Reference	Reference
	Yes	40.2 (33.2–48.6)	47.9 (39.2–58.4)	47.9 (39.3–58.4)
Diabetes^‡^	No	Reference	Reference	Reference
	Yes	4.2 (3.8–4.7)	2.0 (1.8–2.3)	2.0 (1.8–2.3)
Chronic dialysis^‡^	No	Reference	Reference	Reference
	Yes	29.8 (19.5–45.7)	13.8 (8.2–23.1)	13.3 (7.9–22.4)
Transplant^‡^	No	Reference	Reference	Reference
	Yes	17.3 (8.6–35.0)	3.0 (1.3–7.0)	3.3 (1.4–7.8)

† Age at the date of the TB diagnosis for the person with TB disease, and the same date for each matched control.

* Time from cancer to the date of diagnosis of TB for the person with TB disease, and time from cancer to the TB diagnosis date of the matched case for each control.

‡ Medical comorbidities identified during the lookback period for cases and controls.

cOR = crude odds ratio; CI = confidence interval; aOR = adjusted OR.

**Table 4.** Conditional logistic regression for odds of diagnosis of TB associated with prior exposure to cancer (stratified by cancer type), adjusted for demographic characteristics and medical comorbidities.

Variable		cOR (95% CI)	Adjusted (for solid and haematologic cancers) aOR (95% CI)	Adjusted (for all cancer sub-types) aOR (95% CI)
Cancer type	No cancer	Reference	Reference	Reference
	Solid*	11.2 (9.4–13.4)	5.6 (4.6–6.7)	—
	Respiratory	110.6 (71.1–172.2)	—	47.7 (29.0–78.4)
	Head and neck	22.0 (10.2–47.4)	—	11.0 (4.7–25.7)
	Gastrointestinal	14.9 (9.8–22.7)	—	5.9 (3.7–9.2)
	Other solid	9.5 (6.5–14.0)	—	4.9 (3.2–7.5)
	Male genital system	7.1 (4.1–12.5)	—	2.2 (1.2–4.0)
	Gynaecological	2.5 (1.1–5.6)	—	1.9 (0.81–4.6)
	Breast	2.6 (1.3–5.1)	—	1.8 (0.90–3.6)
	Haematologic*	31.1 (20.8–46.5)	15.1 (9.6–24.0)	—
	Other haematologic	57.1 (26.7–122.1)	—	20.8 (8.6–50.6)
	Leukaemia	39.2 (16.1–95.2)	—	20.5 (7.6–55.1)
	Lymphoma	20.9 (11.8–37.3)	—	11.5 (6.0–22.0)
Sex	Female	Reference	Reference	Reference
	Male	1.3 (1.2–1.4)	1.3 (1.3–1.4)	1.3 (1.2–1.4)
Age group, years	0–14	0.63 (0.54–0.74)	0.62 (0.54–0.73)	0.63 (0.54–0.73)
	15–24	Reference	Reference	Reference
	25–34	0.99 (0.88–1.1)	0.94 (0.83–1.1)	0.94 (0.84–1.1)
	35–44	0.92 (0.82–1.0)	0.83 (0.74–0.94)	0.83 (0.74–0.94)
	45–54	1.0 (0.90–1.2)	0.89 (0.78–1.0)	0.89 (0.78–1.0)
	55–64	1.9 (1.7–2.1)	1.6 (1.4–1.9)	1.6 (1.4–1.9)
	65–74	4.2 (3.7–4.7)	3.5 (3.0–3.9)	3.4 (3.0–3.9)
	≥75	12.3 (10.9–14.0)	10.5 (9.2–11.9)	10.4 (9.1–11.8)
HIV	No	Reference	Reference	Reference
	Yes	40.2 (33.2–48.6)	47.9 (39.2–58.4)	48.2 (39.5–58.9)
Diabetes	No	Reference	Reference	Reference
	Yes	4.2 (3.8–4.7)	2.0 (1.8–2.3)	2.0 (1.8–2.3)
Chronic dialysis	No	Reference	Reference	Reference
	Yes	29.8 (19.5–45.7)	14.0 (8.4–23.4)	14.7 (8.7–24.6)
Transplant	No	Reference	Reference	Reference
	Yes	17.3 (8.6–35.0)	2.8 (1.2–6.6)	2.8 (1.2–6.5)

* For cancer type in the unadjusted models, one model included no cancer, solid, and haematologic cancers (excluding the solid and haemotologic sub-types); and another model included no cancer and all other solid and haemotologic cancer sub-types (excluding the broad solid and haematologic groups).

cOR = crude odds ratio; CI = confidence interval; aOR = adjusted OR.

## DISCUSSION

In this study, we found that within 2 years of cancer diagnosis, there was approximately a six-fold increase in the risk of diagnosis of TB disease. Risk varied by cancer type and was very high for respiratory, haematological, and head and neck cancers and moderate for gastrointestinal tract and male genital system tumours. The greatest risk of TB diagnosis occurred within 3 months of cancer diagnosis, with more than half of people with both TB and cancer diagnosed with TB within 3 months of cancer. However, about 40% of people with both TB and cancer were diagnosed with TB more than 6 months following cancer diagnosis, with TB thus considered potentially preventable.

Our findings are consistent with previous studies that found the highest risk of TB disease in individuals with cancer occurs with lung cancer and haematological malignancies.^9,10,24,25^ In the United States, a systematic review and meta-analysis found a strong association between cancer and TB disease, with the highest risk of TB for haematologic (incidence rate ratio [IRR] 26, 95% CI 20–34), head and neck (IRR 16, 95% CI 10–25), and lung cancers (IRR 9, 95% CI 4–20). In that study, the risk of haematologic cancers was three-fold higher than solid tumours (IRR 26 vs 7), similar to our study where the aOR of haematologic cancers was 15.1 compared to 5.6 for solid tumours.^9^ The median follow-up time for included studies was not reported. A cohort study from British Columbia, with a median follow-up of 5 years, found the highest risk of TB diagnosis in those with lung (adjusted hazard ratio [aHR] 11.2, 95% CI 7.4–16.9), sarcoma (aHR 8.1, 95% CI 3.3–19.5), haematologic (leukaemia: aHR 5.6, 95% CI 3.1–10.2; lymphoma: aHR 4.9, 95% CI 2.7–8.7) and gastrointestinal (aHR 2.7, 95% CI 1.7–4.4) cancers.^10^ Lower overall rates for each cancer may be due to the longer follow-up time. Cohort studies from Denmark and Taiwan have also shown strong associations between cancer and TB, with variations in cancer-specific risk likely related to differences between study settings, TB incidence in the country of study, years of follow-up, and cancer treatments.^5,6,8,26^ In 2022, estimated incidences of TB and age-standardised cancer in Canada were respectively 5.7 and 345.9/100,000 population, compared to 4 and 374.7/100,000 in Denmark, and 28 and 335.7/100,000 (in 2017) in Taiwan.^27–30^

We found that the risk of TB disease was highest closer to the time of cancer diagnosis and greatest in the first 6 months post-cancer diagnosis. Contributing factors may include shared diagnostic testing and immunosuppression from active malignancy and cancer therapies which may lead to TB reactivation early in the disease course.^9^ Cancer can lead to immunocompromise due to deficiencies in cell-mediated immunity from the disease or immunosuppressive therapies.^5^ Immunosuppression from cancer can occur through mechanisms including infiltration of lymphoid organs and bone marrow with cancer and administration of chemotherapeutic drugs that can induce immune cell cytopenias.^31^ Cancer-associated weight loss and malnutrition may also increase TB disease risk.^31,32^

Co-prevalent disease was particularly evident for TB and respiratory cancer. This supports findings from British Columbia, where 57.1% of TB diagnoses among people with lung cancer occurred within 6 months of cancer diagnosis.^10^ Lung cancer and pulmonary TB share certain initial clinical and radiological features, and investigations completed for either condition may reveal a concurrent diagnosis of the other.^33^ This may also increase the likelihood of diagnosing subclinical or incipient TB, which might otherwise only have been diagnosed later or never.

A bidirectional relationship that increases the likelihood of co-occurrence of lung cancer and pulmonary TB has been previously described.^34^ Lung cancer increases the risk of TB reactivation through the invasion of old TB granulomas, and cancer treatments may be associated with systemic immunocompromise.^34^ In turn, prior TB disease has been associated with an increased risk of developing cancer, with a 1.6-fold increase in the risk of all cancers and an approximately three-fold increase in the risk of lung cancer in individuals with a history of TB disease.^35^ Hypothesised mechanisms leading to increased risk of cancer following TB disease include chronic inflammation and fibrosis that can promote genetic mutations in lung parenchymal cells;^32,36–38^ and increased production of cytokines such as tumour necrosis factor-alpha which may promote tumour development through angiogenesis, production of genotoxic molecules such as reactive oxygen species, and induction of genes encoding anti-apoptotic molecules.^32,34^

Our higher risk estimates compared to prior studies may be due to our shorter study period (1–2 years, compared to 5–12 years in prior studies reporting follow-up duration).^6,8,10,26^ Previous studies stratifying TB risk by time since cancer have also demonstrated the highest risk closer to the time of cancer diagnosis. A cohort study conducted in Denmark found higher risk within the first year of cancer diagnosis (aHR 4.14, 95% CI 2.88–5.96) compared to 1–5 years post-cancer diagnosis (aHR 1.70, 95% 1.24–2.33).^6^ Similarly, cohort studies from South Korea and Taiwan found the highest risk within the first 6 months of cancer diagnosis, followed by 6–12 months post-diagnosis.^4,26^ We consider 2 years to be a clinically relevant duration, as it encompasses the period of active malignancy and administration of cancer therapies for most patients with cancer, and therefore presents an important period of increased risk that TB preventive strategies may target. While TB diagnoses occurring within 6 months of cancer are unlikely to be preventable, those occurring 6–24 months post-cancer present opportunities for intervention through latent TB screening and treatment at the time of cancer diagnosis.

Our study highlights opportunities for improving TB screening among people with cancer. We identified an overall association between cancer and TB disease, but people with respiratory, haematologic, and head and neck cancers were at the highest risk, and screening should be strongly considered in the presence of these cancer types. In addition, medical comorbidities such as HIV, chronic kidney disease, transplant, immunosuppression, and diabetes increase the risk of TB disease,^12^ and co-occurrence of these conditions with cancer further support screening.

Study strengths include the large population cohort, including all people with TB disease notified in Quebec over a 25-year period, with cancer ascertainment through a cancer registry. However, our study had limitations. We used health administrative databases, which may introduce inaccuracies using ICD codes to identify medical comorbidities. We lacked data on variables that may affect the relationship between cancer and TB disease, including smoking status, alcohol use, obesity, cancer treatments, and socioeconomic status. In particular, smoking is an important risk factor for TB and lung cancer,^31^ and likely contributes to the observed association. Our study population was relatively younger and thus less representative of the broader population, as reflected in the cancer types we identified. However, we accounted for this by adjusting for age and medical comorbidities that may be influenced by age (including adjustment for continuous age and age-squared in sensitivity analyses, Supplementary Data 4/5).

## CONCLUSIONS

We observed a strong association between diagnoses of cancer and TB disease. Our findings suggest a critical period of increased risk of TB disease in the 24 months following cancer diagnosis, with approximately 60% of TB diagnoses occurring within 6 months post-cancer diagnosis. In the absence of TB disease, screening and treatment for potentially preventable TB should be considered, particularly in those with respiratory, hematologic, and head and neck cancers.

## Supplementary Material


