# Supplementary material for: Risk of TB disease in individuals with cancer
Source: IJTLD Open. 2025 Jan 1;2(1):45–52. doi: 10.5588/ijtldopen.24.0440 (PMC11724530; doi:10.5588/ijtldopen.24.0440)
Supplement: Supplementary file 1 [file ijtldopen24-0440_supplementarydata1.docx]

## **SUPPLEMENTARY 1: MEDICAL COMORBIDITY DEFINITIONS**

**Diabetes** (1,2)

[≥ 1 hospital discharge records OR (≥ 2 physician billing records within 2 years^) with an ICD diagnosis code for diabetes mellitus OR 1 prescription for an anti-diabetic medication]

- ICD-9 codes: 250.x
- ICD-10 codes: E10.x, E11.x, E12.x, E13.x, E14.x

**HIV** (3, 4)

[≥ 2 physician billing records OR ≥ 1 hospital discharge record with an ICD code for HIV/AIDS within 2 years] OR [≥ 2 physician billing records with an ICD code for HIV/AIDS OR ≥ 2 dispensation records of HIV medications within 2 years]

- ICD-9 codes: 042, 043, 044, V08
- ICD-10 codes: B20, B21, B22, B23, B24, R75, Z21

**Chronic dialysis** (5-8)

Any peritoneal dialysis OR 2+ codes for haemodialysis at least 91 days apart, with at least one code per week during the intervening period

Peritoneal dialysis codes

- RAMQ physician billing specialistes codes: 09259, 09260, 09291, 15035, 15036
- RAMQ physician billing omnipracticiens codes: 00283-00286
- CCADTC/CCP: 6698
- CCI codes: 1OT53DATS, 1OT53HATS, 1OT53LATS, 1PZ21HPD4, 1SY55LAFT

Haemodialysis codes

- RAMQ physician billing specialistes codes: 09216-09219, 09261-09264, 09274, 09275, 09279, 15040-15048, 15050, 15051, 15457, 15722, 15723
- RAMQ physician billing omnipracticiens codes: 00147, 00287-00290
- ICD-9 codes: V451, V560, V568
- ICD-10 codes: T824, Y602, Y612, Y622, Y841, Z490, Z491, Z492, Z992
- CCADTC/CCP codes: 5127, 5142, 5143, 5195
- CCI codes: 1PZ21HQBR, 1PZ21HQBS, 7SC59QD, 1KG76MZXXA, 1KG76MZXXN, 1KY76

**Solid organ transplant** (9)

≥ 1 procedure code in hospital discharge records for transplant:

- CCADTC/CCP codes: 4559, 4569, 4959, 5309, 5352, 6249, 6481, 6483, 6484, 6759, 7792
- CCI codes: 1GR85, 1GT85, 1HY85, 1HZ85, 1NK85, 1NP85, 1OA85, 1OB85, 1OJ85, 1OK85, 1PC85, 1RB85, 1WY19

**Abbreviations**CCADTC/CCP: Classification canadienne des actes diagnostiques, thérapeutiques et chirurgicaux / Canadian Classification of Diagnostic, Therapeutic, and Surgical Procedures
CCI: Canadian Classification of Health Interventions
ICD-9: International Classification of Diseases, 9^th^ Revision, Quebec
ICD-10: International Statistical Classification of Diseases and Related Health Problems, 10^th^ Revision, Canada
RAMQ: Régie de l’assurance maladie du Québec

References:

1. Lipscombe LL, Hwee J, Webster L, Shah BR, Booth GL, Tu K. Identifying diabetes cases from administrative data: a population-based validation study. BMC health services research. 2018;18(1):316.

2. Wang F, Stewart M, McDermott S, Kazanjian A, Vissandjee B, DesMeules M, et al. Migration and diabetes in British Columbia and Quebec: prevalence and health service utilization. Can J Public Health. 2012;103(1):59-64.

3. Antoniou T, Zagorski B, Loutfy MR, Strike C, Glazier RH. Validation of case-finding algorithms derived from administrative data for identifying adults living with human immunodeficiency virus infection. PloS one. 2011;6(6):e21748.

4. Nosyk B, Colley G, Yip B, Chan K, Heath K, Lima VD, et al. Application and validation of case-finding algorithms for identifying individuals with human immunodeficiency virus from administrative data in British Columbia, Canada. PloS one. 2013;8(1):e54416.

5. Clement FM, James MT, Chin R, Klarenbach SW, Manns BJ, Quinn RR, et al. Validation of a case definition to define chronic dialysis using outpatient administrative data. BMC Med Res Methodol. 2011;11:25.

6. Grams ME, Plantinga LC, Hedgeman E, Saran R, Myers GL, Williams DE, et al. Validation of CKD and related conditions in existing data sets: A systematic review. Am J Kidney Dis. 2011;57(1):44-54.

7. Ronksley PE, Tonelli M, Quan H, Manns BJ, James MT, Clement FM, et al. Validating a case definition for chronic kidney disease using administrative data. Nephrol Dial Transplant. 2012;27(5):1826-31.

8. Shah M, Avgil Tsadok M, Jackevicius CA, Essebag V, Eisenberg MJ, Rahme E, et al. Warfarin use and the risk for stroke and bleeding in patients with atrial fibrillation undergoing dialysis. Circulation. 2014;129(11):1196-203.

9. Lam NN, McArthur E, Kim SJ, Knoll GA. Validation of kidney transplantation using administrative data. Can J Kidney Health Dis. 2015;2:20.

## **SUPPLEMENTARY 2: CATEGORIZATION OF CANCERS BY ICD-O-3 CODES**

| Category | Sub-categories | ICD-O-3 codes |
| --- | --- | --- |
| Head and neck |  | C00-14x: Lip, oral cavity and pharynx  C30-31x: Nasal cavity, middle ear and accessory sinuses  32x: Larynx |
| Respiratory | Lung | C34x: Bronchus and lung |
|  | Other respiratory system | C33x: Trachea  C38.1-9: Mediastinum and pleura  C39x: Other and ill-defined sites within respiratory system and intrathoracic organs |
| Breast |  | C50x: Breast |
| Gastrointestinal | Esophagus | C15x: Esophagus |
|  | Stomach | C16x: Stomach |
|  | Colorectal | C18-20x: Colon, rectosigmoid junction, rectum  C26.0: Intestinal tract, NOS |
|  | Liver | C22.0: Liver |
|  | Pancreas | C25x: Pancreas |
|  | Other digestive system | C17x: Small intestine  C21x: Anus and anal canal  C22.1: Intrahepatic bile duct  C23x: Gallbladder  C24x: Other and unspecified parts of biliary tract  C26.8-9: Overlapping lesion of digestive system  C48x: Gastrointestinal tract, NOS |
| Gynecological | Cervix | C53x: Cervix uteri |
|  | Uterus | C54x: Corpus uteri  C55x: Uterus, NOS |
|  | Ovary | C56.9: Ovary |
|  | Other | C51x: Vulva  C52x: Vagina  C57x: Other and unspecified female genital organs  C58x: Placenta |
| Male genital system | Prostate | C61.9: Prostate gland |
|  | Testis | C62x: Testis |
|  | Other | C60x: Penis  C63x: Other and unspecified male genital organs |
| Other solid | **Urinary system** | |
|  | Kidney and renal pelvis | C64.9: Kidney  C65.9: Renal pelvis |
|  | Bladder | C67x: Bladder |
|  | Other | C66x: Ureter  C68x: Other and unspecified urinary organs |
|  | **Brain/CNS** | C70-72x: Meninges, brain, spinal cord, cranial nerves and other parts of central nervous system |
|  | **Endocrine** | |
|  | Thyroid | C73x: Thyroid gland |
|  | Other | C37.9: Thymus  C74x: Adrenal gland  C75x: Other endocrine glands and related structures |
|  | **Skin** | |
|  | Melanoma | C44x: Skin AND Type: 8720-8790: Nevi and melanomas |
|  | Other (not included) | C44x excl. 8720-8790 (see above) |
|  | **Other** | |
|  | Bone and joints | C40-41x: Bones, joints and articular cartilage |
|  | Soft tissue | C38.0: Heart  C47x: Peripheral nerves and autonomic nervous system  C49x: Connective, subcutaneous and other soft tissues |
|  | Eye | C69x: Eye and adnexa |
|  | Other, ill-defined, and unknown | C76x: Other and ill-defined sites  C80x: Unknown primary site |
| Hematologic | Leukemia | Type:  9733: Plasma cell leukemia  9742: Mast cell leukemia  9800-9801, 9805-9809 : Leukemia, NOS  9820 : Lymphoid leukemia, NOS  9826 : Burkitt cell leukemia  9831: T-cell large granular lymphocytic leukemia  9836: Precursor B-cell lymphoblastic leukemia  9840, 9860-9861, 9863, 9865-9867, 9869-9876, 9891, 9895-9898, 9910, 9911, 9920, 9930-9931, 9940, 9945-9946, 9948: Myeloid leukemias  9963-9964: chronic neutrophilic leukemia, chronic eosinophilic leukemia, NOS  **For sites C42.0, .1, .4:** Type 9811-9818: B lymphoblastic leukemia/lymphoma  9823: B-cell chronic lymphocytic leukemia/small lymphocytic lymphoma  9827: Adult T-cell leukemia/lymphoma  9837: Precursor T-cell lymphoblastic leukemia/lymphoma |
|  | Lymphoma | Type 9650-9667: Hodgkin lymphoma  Type 9590-9597: Malignant lymphomas, NOS or diffuse  9670-9719, 9724-9729: Non-hodgkin lymphoma  9735: Plasmablastic lymphoma  9737: ALK positive large B-cell lymphoma  9738: Large B-cell lymphoma arising in HHV8-associated multicentric Castleman disease  **For all sites except C42.0, .1, .4:** Type 9811-9818: B lymphoblastic leukemia/lymphoma  9823: B-cell chronic lymphocytic leukemia/small lymphocytic lymphoma  9827: Adult T-cell leukemia/lymphoma  9837: Precursor T-cell lymphoblastic leukemia/lymphoma |
|  | Multiple myeloma | **For site C42.1:**  Type 9731: Plasmacytoma  9732: Multiple myeloma  9734: Plasmacytoma, extramedullary |
|  | Other hematologic | 9740-9741: Mast cell tumors excluding mast cell leukemia  9750, 9751, 9755 – 9759: Neoplasms of histiocytes and accessory lymphoid cells  9760-9762, 9764, 9767, 9768: Malignant Immunoproliferative diseases  9950-9962, 9965-9967: Chronic myeloproliferative disorders (except chronic neutrophilic leukemia and chronic eosinophilic leukemia)  9970, 9971, 9975: Other hematologic disorders  9980, 9982-9987, 9989, 9991, 9992: Myelodysplastic syndromes |

References

1. Xie L, Semenciw R, Mery L. Cancer incidence in Canada: trends and projections (1983-2032). Health promotion and chronic disease prevention in Canada : research, policy and practice. 2015;35 Suppl 1:2-186.

2. Canadian Cancer Statistics Advisory in collaboration with the Canadian Cancer Society SCatPHAoC. Canadian Cancer Statistics: A 2022 special report on cancer prevalence. Toronto, ON: Canadian Cancer Society; 2022.

## **SUPPLEMENTARY 3: CANCER SUB-TYPES FOR ALL PEOPLE WITH TB DISEASE AND CONTROLS**

|  | Overall | |
| --- | --- | --- |
|  | **TB disease**  **(n = 4,283)** | **Controls**  **(n = 268,420)** |
| No cancer | **4,091 (95.5)** | **267,498 (99.7)** |
| Solid cancer types | **155 (3.6)** | **850 (0.3)** |
| Breast | 9 (0.2) | 210 (0.1) |
| Gastrointestinal | 28 (0.7) | 115 (0.0) |
| Gynecological | 6 (0.1) | 153 (0.1) |
| Head and neck | 9 (0.2) | 25 (0.0) |
| Male genital system | 14 (0.3) | 116 (0.0) |
| Other solid | 30 (0.7) | 198 (0.1) |
| Respiratory* | 59 (1.4) | 33 (0.0) |
| Hematologic cancer types | **37 (0.9)** | **72 (0.0)** |
| Leukemia | 8 (0.2) | 13 (0.0) |
| Lymphoma | 16 (0.4) | 45 (0.0) |
| Other hematologic* | 13 (0.3) | 14 (0.0) |

TB: tuberculosis

*respiratory includes cancers coded as bronchus and lung, trachea, mediastinum and pleura, other and ill-defined sites within respiratory system and intrathoracic organs; other hematologic includes multiple myeloma, mast cell tumors excluding mast cell leukemia, neoplasms of histiocytes and accessory lymphoid cells, malignant immunoproliferative diseases, chronic myeloproliferative disorders (except chronic neutrophilic leukemia and chronic eosinophilic leukemia), other hematologic disorders, myelodysplastic syndromes

## **SUPPLEMENTARY 4: MODELS WITH AGE ADJUSTED AS A CONTINUOUS VARIABLE**

Conditional logistic regression for odds of diagnosis of TB associated with prior exposure to cancer, adjusted for demographic characteristics and medical comorbidities as shown in the table.

|  | | Unadjusted | Adjusted (for cancer) | Adjusted (for time from cancer to TB) |
| --- | --- | --- | --- | --- |
| Variable | | **cOR (95% CI)** | **aOR (95% CI)** | **aOR (95% CI)** |
| Cancer | No | Reference | Reference | - |
|  | Yes | 12.8 (10.9-15.0) | 6.6 (5.6-7.9) | - |
| Time from cancer to TB diagnosis^ | No cancer | Reference | - | Reference |
|  | Up to 3 months | 53.1 (40.6-69.5) | - | 25.2 (18.8-33.6) |
|  | 4-6 months | 6.8 (3.9-11.9) | - | 3.9 (2.2-7.0) |
|  | 7-12 months | 9.3 (6.4-13.3) | - | 5.1 (3.5-7.4) |
|  | 13-18 months | 4.9 (3.1-7.9) | - | 2.4 (1.4-3.9) |
|  | 19-24 months | 6.4 (4.2-9.9) | - | 3.4 (2.2-5.4) |
| Sex | Female | Reference | Reference | Reference |
|  | Male | 1.3 (1.2-1.4) | 1.3 (1.2-1.4) | 1.3 (1.2-1.4) |
| Age* |  | 1.0 (1.0-1.0) | 1.0 (1.0-1.0) | 1.0 (1.0-1.0) |
| HIV^%^ | No | Reference | Reference | Reference |
|  | Yes | 40.2 (33.2-48.6) | 37.0 (30.4-45.1) | 37.1 (30.4-45.2) |
| Diabetes^%^ | No | Reference | Reference | Reference |
|  | Yes | 4.2 (3.8-4.7) | 2.2 (2.0-2.5) | 2.2 (2.0-2.5) |
| Chronic dialysis^%^ | No | Reference | Reference | Reference |
|  | Yes | 29.8 (19.5-45.7) | 13.6 (8.3-22.3) | 13.3 (8.1-21.9) |
| Transplant^%^ | No | Reference | Reference | Reference |
|  | Yes | 17.3 (8.6-35.0) | 2.4 (1.1-5.6) | 2.7 (1.2-6.2) |

aOR: adjusted odds ratio; CI: confidence interval; cOR: crude odds ratio; HIV: human immunodeficiency virus; TB: tuberculosis

*age at the date of the TB diagnosis for the person with TB disease, and the same date for each matched control; ^time from cancer to the date of diagnosis of TB for the person with TB disease, and time from cancer to the TB diagnosis date of the matched case for each control; ^%^medical comorbidities identified during the lookback period for cases and controls

Conditional logistic regression for odds of diagnosis of TB associated with prior exposure to cancer (stratified by cancer type), adjusted for demographic characteristics and medical comorbidities as shown in the table.

| Variable |  | Unadjusted | Adjusted (for solid and hematologic cancers) | Adjusted (for all cancer sub-types) |
| --- | --- | --- | --- | --- |
|  |  | **cOR (95% CI)** | **aOR (95% CI)** | **aOR (95% CI)** |
| Cancer type | No cancer | Reference | Reference | Reference |
|  | Solid* | 11.2 (9.4-13.4) | 5.9 (4.9-7.0) | - |
|  | Respiratory | 110.6 (71.1-172.2) | - | 46.7 (29.2-74.5) |
|  | Head and neck | 22.0 (10.2-47.4) | - | 10.9 (4.9-24.3) |
|  | Gastrointestinal | 14.9 (9.8-22.7) | - | 6.8 (4.4-10.5) |
|  | Other solid | 9.5 (6.5-14.0) | - | 5.1 (3.4-7.7) |
|  | Male genital system | 7.1 (4.1-12.5) | - | 2.7 (1.5-4.7) |
|  | Gynecological | 2.5 (1.1-5.6) | - | 1.8 (0.8-4.3) |
|  | Breast | 2.6 (1.3-5.1) | - | 1.7 (0.84-3.3) |
|  | Hematologic* | 31.1 (20.8-46.5) | 15.9 (10.3-24.6) | - |
|  | Other hematologic | 57.1 (26.7-122.1) | - | 22.8 (10.0-51.9) |
|  | Leukemia | 39.2 (16.1-95.2) | - | 22.9 (8.9-58.9) |
|  | Lymphoma | 20.9 (11.8-37.3) | - | 11.4 (6.1-21.4) |
| Sex | Female | Reference | Reference | Reference |
|  | Male | 1.3 (1.2-1.4) | 1.3 (1.2-1.4) | 1.3 (1.2-1.4) |
| Age |  | 1.0 (1.0-1.0) | 1.0 (1.0-1.0) | 1.0 (1.0-1.0) |
| HIV | No | Reference | Reference | Reference |
|  | Yes | 40.2 (33.2-48.6) | 37.0 (30.3-45.1) | 37.5 (30.7-45.7) |
| Diabetes | No | Reference | Reference | Reference |
|  | Yes | 4.2 (3.8-4.7) | 2.2 (2.0-2.5) | 2.2 (2.0-2.5) |
| Chronic dialysis | No | Reference | Reference | Reference |
|  | Yes | 29.8 (19.5-45.7) | 13.8 (8.4-22.5) | 14.5 (8.8-23.8) |
| Transplant | No | Reference | Reference | Reference |
|  | Yes | 17.3 (8.6-35.0) | 2.3 (1.0-5.3) | 2.2 (0.96-5.2) |

aOR: adjusted odds ratio; CI: confidence interval; cOR: crude odds ratio; HIV: human immunodeficiency virus; TB: tuberculosis

*For cancer type in the unadjusted models, one model included no cancer, solid, and hematologic cancers (excluding the solid and hematologic sub-types); and another model included no cancer and all other solid and hematologic cancer sub-types (excluding the broad solid and hematologic groups).

## **SUPPLEMENTARY 5: MODELS WITH AGE AND AGE-SQUARED ADJUSTED AS CONTINUOUS VARIABLES**

Conditional logistic regression for odds of diagnosis of TB associated with prior exposure to cancer, adjusted for demographic characteristics and medical comorbidities as shown in the table.

|  | | Unadjusted | Adjusted (for cancer) | Adjusted (for time from cancer to TB) |
| --- | --- | --- | --- | --- |
| Variable | | **cOR (95% CI)** | **aOR (95% CI)** | **aOR (95% CI)** |
| Cancer | No | Reference | Reference | - |
|  | Yes | 12.8 (10.9-15.0) | 6.3 (5.3-7.6) | - |
| Time from cancer to TB diagnosis^ | No cancer | Reference | - | Reference |
|  | Up to 3 months | 53.1 (40.6-69.5) | - | 25.4 (18.8-34.5) |
|  | 4-6 months | 6.8 (3.9-11.9) | - | 3.9 (2.2-7.0) |
|  | 7-12 months | 9.3 (6.4-13.3) | - | 4.7 (3.2-7.0) |
|  | 13-18 months | 4.9 (3.1-7.9) | - | 2.1 (1.2-3.5) |
|  | 19-24 months | 6.4 (4.2-9.9) | - | 3.3 (2.1-5.2) |
| Sex | Female | Reference | Reference | Reference |
|  | Male | 1.3 (1.2-1.4) | 1.3 (1.3-1.4) | 1.3 (1.3-1.4) |
| Age* |  | 1.0 (1.0-1.0) | 0.95 (0.95-0.96) | 0.95 (0.95-0.96) |
| Age-squared* |  | 1.0 (1.0-1.0) | 1.0 (1.0-1.0) | 1.0 (1.0-1.0) |
| HIV^%^ | No | Reference | Reference | Reference |
|  | Yes | 40.2 (33.2-48.6) | 46.6 (38.2-56.8) | 46.6 (38.2-56.8) |
| Diabetes^%^ | No | Reference | Reference | Reference |
|  | Yes | 4.2 (3.8-4.7) | 2.0 (1.8-2.2) | 2.0 (1.8-2.2) |
| Chronic dialysis^%^ | No | Reference | Reference | Reference |
|  | Yes | 29.8 (19.5-45.7) | 13.5 (8.1-22.6) | 13.1 (7.8-21.9) |
| Transplant^%^ | No | Reference | Reference | Reference |
|  | Yes | 17.3 (8.6-35.0) | 2.8 (1.2-6.6) | 3.2 (1.4-7.4) |

aOR: adjusted odds ratio; CI: confidence interval; cOR: crude odds ratio; HIV: human immunodeficiency virus; TB: tuberculosis

*age at the date of the TB diagnosis for the person with TB disease, and the same date for each matched control; ^time from cancer to the date of diagnosis of TB for the person with TB disease, and time from cancer to the TB diagnosis date of the matched case for each control; ^%^medical comorbidities identified during the lookback period for cases and controls

Conditional logistic regression for odds of diagnosis of TB associated with prior exposure to cancer (stratified by cancer type), adjusted for demographic characteristics and medical comorbidities as shown in the table.

| Variable |  | Unadjusted | Adjusted (for solid and hematologic cancers) | Adjusted (for all cancer sub-types) |
| --- | --- | --- | --- | --- |
|  |  | **cOR (95% CI)** | **aOR (95% CI)** | **aOR (95% CI)** |
| Cancer type | No cancer | Reference | Reference | Reference |
|  | Solid* | 11.2 (9.4-13.4) | 5.5 (4.6-6.7) | - |
|  | Respiratory | 110.6 (71.1-172.2) | - | 44.8 (27.5-73.0) |
|  | Head and neck | 22.0 (10.2-47.4) | - | 11.2 (4.9-25.8) |
|  | Gastrointestinal | 14.9 (9.8-22.7) | - | 5.9 (3.8-9.3) |
|  | Other solid | 9.5 (6.5-14.0) | - | 5.0 (3.3-7.7) |
|  | Male genital system | 7.1 (4.1-12.5) | - | 2.2 (1.2-4.1) |
|  | Gynecological | 2.5 (1.1-5.6) | - | 1.9 (0.80-4.5) |
|  | Breast | 2.6 (1.3-5.1) | - | 1.7 (0.85-3.4) |
|  | Hematologic* | 31.1 (20.8-46.5) | 15.6 (9.9-24.6) | - |
|  | Other hematologic | 57.1 (26.7-122.1) | - | 21.2 (8.9-51.0) |
|  | Leukemia | 39.2 (16.1-95.2) | - | 20.9 (7.9-55.1) |
|  | Lymphoma | 20.9 (11.8-37.3) | - | 12.0 (6.3-22.7) |
| Sex | Female | Reference | Reference | Reference |
|  | Male | 1.3 (1.2-1.4) | 1.3 (1.3-1.4) | 1.3 (1.2-1.4) |
| Age |  | 1.0 (1.0-1.0) | 0.95 (0.95-0.96) | 0.95 (0.95-0.96) |
| Age-squared |  | 1.0 (1.0-1.0) | 1.0 (1.0-1.0) | 1.0 (1.0-1.0) |
| HIV | No | Reference | Reference | Reference |
|  | Yes | 40.2 (33.2-48.6) | 46.5 (38.1-56.8) | 47.0 (38.5-57.3) |
| Diabetes | No | Reference | Reference | Reference |
|  | Yes | 4.2 (3.8-4.7) | 2.0 (1.8-2.2) | 2.0 (1.8-2.2) |
| Chronic dialysis | No | Reference | Reference | Reference |
|  | Yes | 29.8 (19.5-45.7) | 13.7 (8.2-22.9) | 14.4 (8.6-24.1) |
| Transplant | No | Reference | Reference | Reference |
|  | Yes | 17.3 (8.6-35.0) | 2.7 (1.2-6.3) | 2.6 (1.1-6.1) |

aOR: adjusted odds ratio; CI: confidence interval; cOR: crude odds ratio HIV: human immunodeficiency virus; TB: tuberculosis

*For cancer type in the unadjusted models, one model included no cancer, solid, and hematologic cancers (excluding the solid and hematologic sub-types); and another model included no cancer and all other solid and hematologic cancer sub-types (excluding the broad solid and hematologic groups).
